# Chitosan and Its Carboxymethyl-Based Membranes Produced by Crosslinking with Magnesium Phytate

**DOI:** 10.3390/molecules28165987

**Published:** 2023-08-10

**Authors:** Adam Zając, Wojciech Sąsiadek, Lucyna Dymińska, Paulina Ropuszyńska-Robak, Jerzy Hanuza, Maciej Ptak, Szymon Smółka, Radosław Lisiecki, Katarzyna Skrzypczak

**Affiliations:** 1 Department of Bioorganic Chemistry, Faculty of Production Engineering, Wroclaw University of Economics and Business, 118-120 Komandorska Str., 53-345 Wrocław, Poland; 2 Institute of Low Temperature and Structure Research, 2 Okólna Str., 50-422 Wrocław, Poland; 3 Faculty of Chemistry, Wrocław University of Science and Technology, 27 Wybrzeże Wyspiańskiego Str., 50-370 Wrocław, Poland

**Keywords:** phytate crosslinked chitosan, synthesis and spectroscopic properties, crosslinking mechanism, new packaging material, mechanical characterization

## Abstract

Membranes produced by crosslinking chitosan with magnesium phytate were prepared using highly deacetylated chitosan and its *N*-carboxymethyl, *O*-carboxymethyl and *N*,*O*-carboxymethyl derivatives. The conditions of the membrane production were described. IR, Raman, electron absorption and emission spectra were measured and analyzed for all the substrates. It was found that *O*-carboxymethyl chitosan derivative is the most effectively crosslinked by magnesium phytate, and the films formed on this substrate exhibit good mechanical parameters of strength, resistance and stability. Strong O–H···O hydrogen bonds proved to be responsible for an effective crosslinking process. Newly discovered membrane types produced from chitosan and magnesium phytate were characterized as morphologically homogenous and uniform by scanning electron microscopy (SEM) and IR measurements. Due to their good covering properties, they do not have pores or channels and are proposed as packaging materials.

## 1. Introduction

Polysaccharide-based membranes have been used for years in medicine, pharmacy, the food industry and technical areas. Polysaccharides are mainly extracted from plants, algae and animal sources. Chitosan is an example of a polysaccharide obtained by deacetylation of chitin, the second most abundant organic compound in nature after cellulose. Due to its biocompatibility, biodegradability, non-toxicity and chemical properties, it was widely used for producing films, gels, nano- and micro-particles and hydrogel beads [1,2,3,4,5,6,7,8,9]. For these reasons, chitosan is considered an attractive material for the preparation of membranes for various applications [10,11,12,13,14,15,16,17,18,19,20]. However, it has been found that chitosan-based hydrogel membranes exhibit low mechanical stability, high water content and a loose three-dimensional arrangement. Bulk and surface crosslinking was proposed as a new method for improving the chemical and mechanical stability of chitosan and prepared from their products [15,16,19,20,21,22,23,24,25,26,27,28,29,30,31]. Different types of crosslinkers are used, both covalently and ionically coupled to chitosan. In these processes glutaraldehyde and glyoxal [22,23,26,32], genipine, citrate, tripolyphosphate and sulfate were applied [32,33]. In other studies, starch [25], montmorillonite [29] and vanillin [30] were used as crosslinkers for chitosan. It was found that the properties of the final products, namely, mechanical stretching, swelling, permeation of low molecular weight molecules and drug releasing [34] depend on the pH of the used media and charge density [35] as well as the degree of deacetylation of chitosan [32]. The effect of the preparation conditions and molecular weight of chitosan on the structure, crystallinity, morphology, hydrophilic properties and water state were studied using several methods [21]. The obtained chitosan-based membranes with different ionic crosslinking densities were proposed for pharmaceutical and industrial applications [21].

In the present work, we propose a new technique of chitosan crosslinking using magnesium phytate. We believe that membranes produced by this process will be applied in the medicine and food industries. The materials we obtained were studied by infrared and Raman spectroscopy methods, including using a confocal Raman microscope for mapping their surface. Electron absorption and emission measurements were also taken. These methods are very sensitive to the presence of structural changes in materials and changes in their chemical composition. In addition, they register the presence of various functional groups in their composite. They play a special role in determining how to link biopolymers with a component in the form of a composite that bonds them by a system of chemical bonds, ionic interactions and hydrogen bridges.

## 2. Results and Discussion

**Figure 1:**
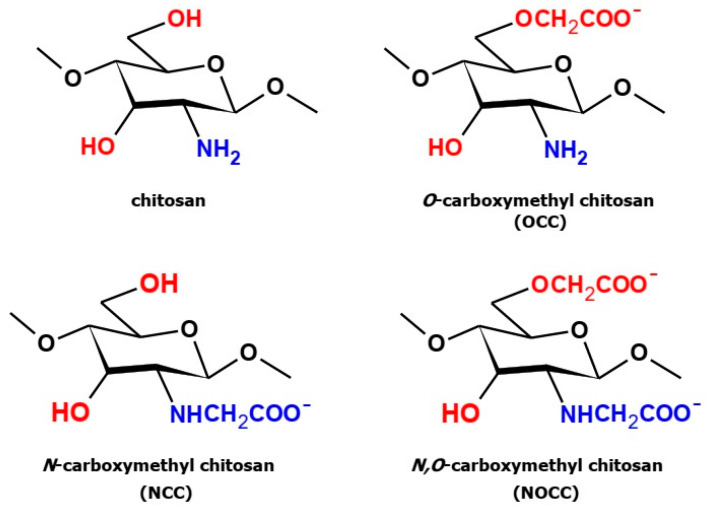
Structures of chitosan and three types of its carboxymethyl derivatives.

Three types of carboxymethyl derivatives of chitosan (Section 3.3), namely, *N*-carboxymethyl chitosan (**NCC**), *O*-carboxymethyl chitosan (**OCC**) and *N*,*O*-carboxymethyl chitosan (**NOCC**), were obtained as the results of the below-described syntheses. The structures of these derivatives are shown in Figure 1. All the obtained products were identified by measuring their IR and Raman spectra and comparing them with the spectra of free chitosan.

### 2.1. Vibrational Spectra

**Figure 2:**
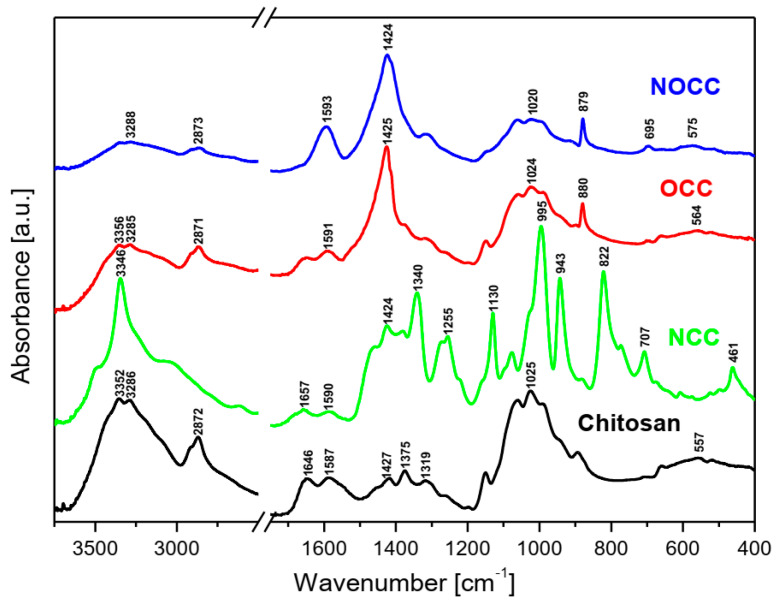
FT-IR spectra of the studied chitosan and its *N*- and *O*-carboxymethyl derivatives.

**Figure 3:**
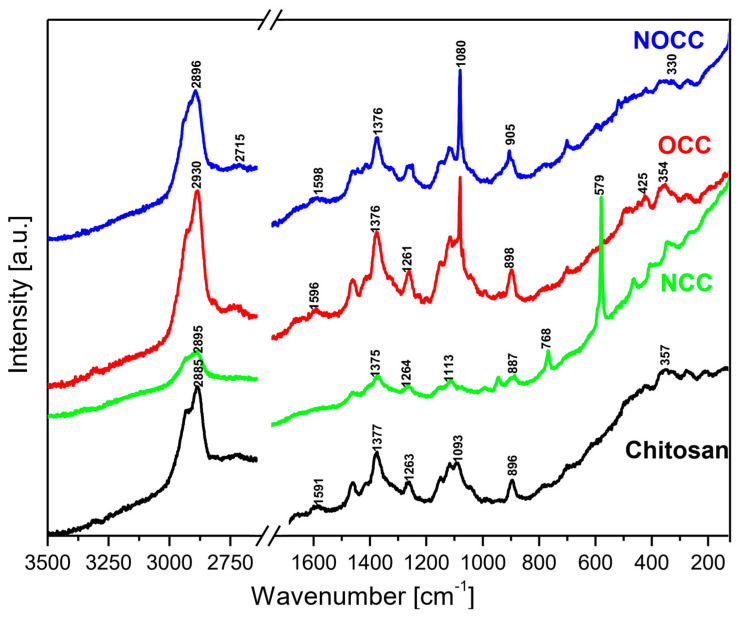
Raman spectra of the studied chitosan and its *N*- and *O*-carboxymethyl derivatives.

The MIR and FIR spectra as well as Raman spectra of the studied chitosan and its derivatives are shown in Figure 2 and Figure 3, respectively.

Analyzing the IR and Raman spectra of the studied materials it should be noted that all spectral patterns are very similar because all the studied compounds contain the same basic skeleton—the pyranoid ring. The positions of vibrational bands corresponding to this unit are practically stable for all the studied samples. The differences between them appears because carboxymethyl derivatives contain an additional –CH_2_-COO– unit substituted at the hydroxyl or amine group. The bands corresponding to this chromophore are observed in the following ranges of the IR spectra: 1590–1593, 1381–1414, 1262–1283, 773–822, 694–605 and 512–575 cm^−1^. The substitution of the carboxymethyl unit at hydroxyl or amine groups provokes specific changes in typical ranges of the IR and Raman spectra. Such characteristic vibrations are observed in the following ranges:

(a) Very strong bands observed at 1080 cm^−1^ in the Raman spectra of **NOCC** and **OCC** derivatives correspond to the symmetric υ_s_(C-O-C) vibration; they originate from the φ-CH_2_-OCH_2_COO fragment of these compounds.
(b) The carboxymethylation of the pyranoid ring at the C-CH_2_-OH substituent also brings about changes in the IR spectra of **OCC** and **NOCC** derivatives—medium intensity bands are observed at 890 and 879 cm^−1^. They vibrations correspond to coupled υ_as_(φ) + δ(CH) + ρ(CH_2_) vibrations of the pyranoid ring and methyl group of the φ-CH_2_-O- substituent.
(c) Other changes are observed in the Raman spectra of the **NCC** and **NOCC** derivatives—new bands observed at 960 and 955 cm^−1^ correspond to the vibrations of the pyranoid ring coupled with the γ(NH) motion.

The abovementioned changes in the IR and Raman spectra should be considered diagnostic effects of the carboxymethylation of the pyranoid ring in chitosan derivatives.

The assignment of the observed wavenumbers to the respective vibrations is proposed on the basis of quantum chemical PED calculations performed for *N*-acetylated chitosan reported in our previous paper [36]. Supplementary Table S1A,B presents these data.

### 2.2. UV–Vis Spectra

**Figure 4:**
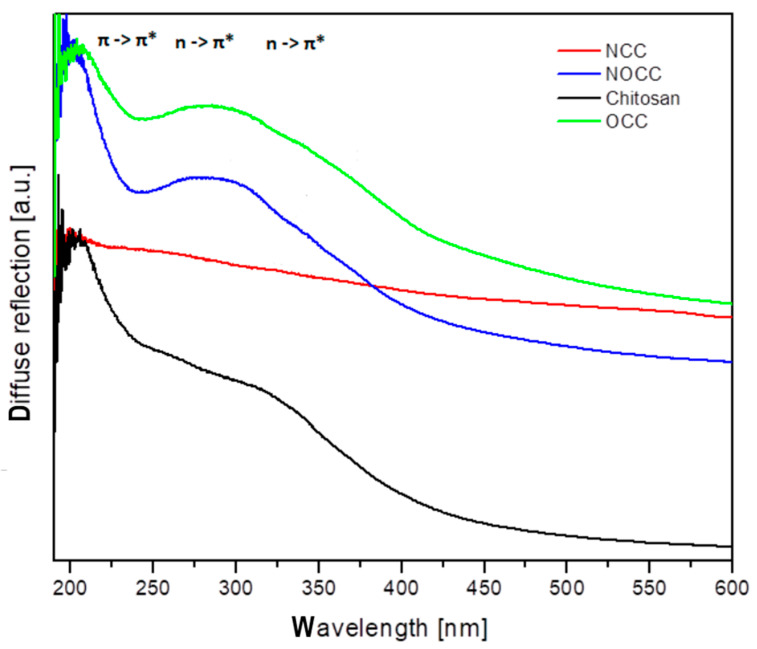
Diffuse reflectance spectra of chitosan and its studied derivatives.

The diffuse reflectance spectra of chitosan and its **NCC**, **OCC** and **NOCC** derivatives measured in the solid state are shown in Figure 4. The spectral patterns observed in the 200–600 nm range exhibit very broad bands with the maxima in the 250–340 nm range. The spectrum of chitosan contains five components observed as shoulders at 255, 286, 312, 340 and 370 nm. The spectra of the **OCC** and **NOCC** derivatives are somewhat different containing a broad band at 286 nm with four shoulders of its slope at 255, 281, 295, 340 and 370 nm. The spectrum of the **NCC** derivative is very weak, probably due to amorphization of this sample. The assignment of the observed bands to the respective electron transitions could be made using the same approximation as for other heterocyclic rings. The singlet states of the pyranoid ring in chitosan should be assigned to the following transitions: S_1_ = 27,027 cm^−1^, S_2_ = 29,411 cm^−1^, S_3_ =32,051 cm^−1^, S_4_ = 34,965 cm^−1^ and S_5_ = 39,215 cm^−1^. The carboxymethylation of chitosan shifts some transitions into the following wavelengths: S_1_ = 27,027 cm^−1^, S_2_ = 29,411 cm^−1^, S_3_ = 33,898 cm^−1^, S_4_ = 35,587 cm^−1^ and S_5_ = 39,215 cm^−1^.

The electron transitions observed in the UV–Vis spectra should be assigned to the π → π* at 200–255 nm and n(Opyranoid) → π* at 255–300 nm, n(Namine) π* at 300–400 nm. These energy states will be compared to those obtained from the emission studies.

### 2.3. Emission Spectra

**Figure 5:**
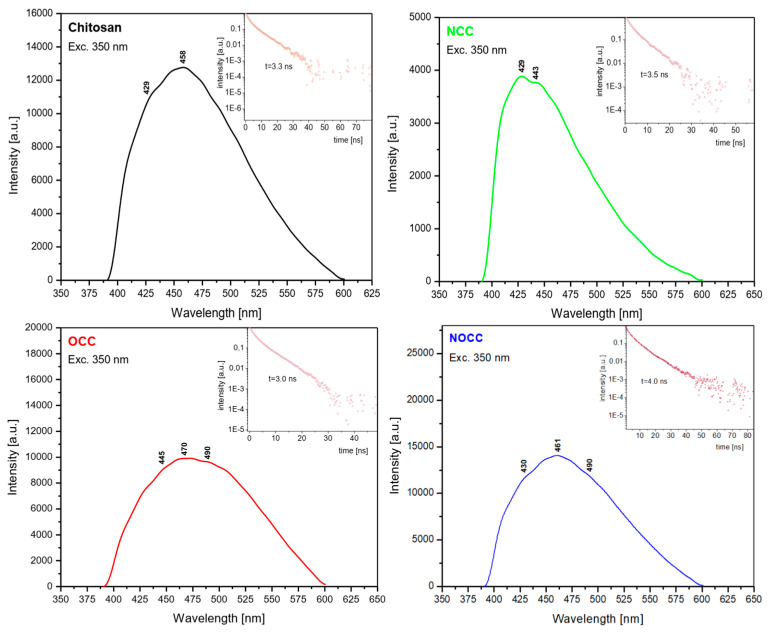
Comparison of the emission spectra of chitosan and its studied derivatives.

The emission spectra of the studied chitosan and its **OCC**, **NCC** and **NOCC** derivatives were measured as solids using excitation at 350 nm (28,571 cm^−1^); they are shown in Figure 5. All the spectra contain a very broad band observed in the 400–600 nm range with the maxima at 458 nm (21,834 cm^−1^) for chitosan, 429 nm (23,310 cm^−1^) for **NCC**, 470 nm (21,276 cm^−1^) for **OCC** and 461 nm (21,691 cm^−1^) for the **NOCC** derivative. On the slope of these bands 2–3 shoulders are observed showing that they have a triplet structure. They are observed at the following wavenumbers: 23,410 and 19,607 cm^−1^ for chitosan, 22,573 and 20,000 cm^−1^ for **NCC**, 33,471 and 20,408 cm^−1^ for **OCC** and 23,255 and 20,408 cm^−1^ for the **NOCC** derivative.

The energy gap between the electron absorption and emission studies (Stokes shift) can be calculated for each studied compound. It is equal to 12,648 cm^−1^ for chitosan, 13,689 cm^−1^ for the **OCC** derivative and 13,896 cm^−1^ for the **NOCC** one. Such energy gaps of the 12,600–13,900 cm^−1^ order could not be explained as having derived from simple S_1_ → S_0_ emission. The emission transitions are excited at 28,571 cm^−1^, i.e., slightly above the S_1_ level, but the emissions occur from the levels located in the 19,400–23,260 cm^−1^ range. The explanation of this behavior is that the depopulation mechanism of the excited states in chitosan derivatives proceeds by the intersystem crossing of the singlet states to triplet levels S_0_ → S_1_ → T_1,2,3_. On the basis of the measured emission spectra, the following succession of the triplet states in the studied compounds should be proposed (Table 1).

The small energetic splitting between the T_1_, T_2_ and T_3_ states causes overlapping of these levels in the form of a broad spectral band in the emission spectra. The participation of the triplet levels in the depopulation of the excited states of the chitosan derivatives is confirmed by the measurements of their lifetimes. They are 3.3 ns for chitosan, 3.6 ns for **NCC**, 3.0 ns for **OCC** and 4.0 ns for **NOCC**. They are very short, characteristic for these types of electron processes.

### 2.4. Spectral and Microscopic Studies of the Crosslinked Chitosan

**Figure 6:**
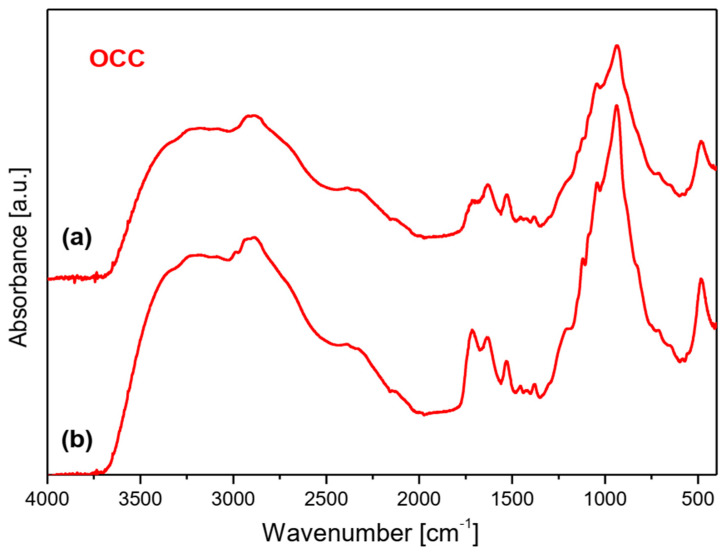

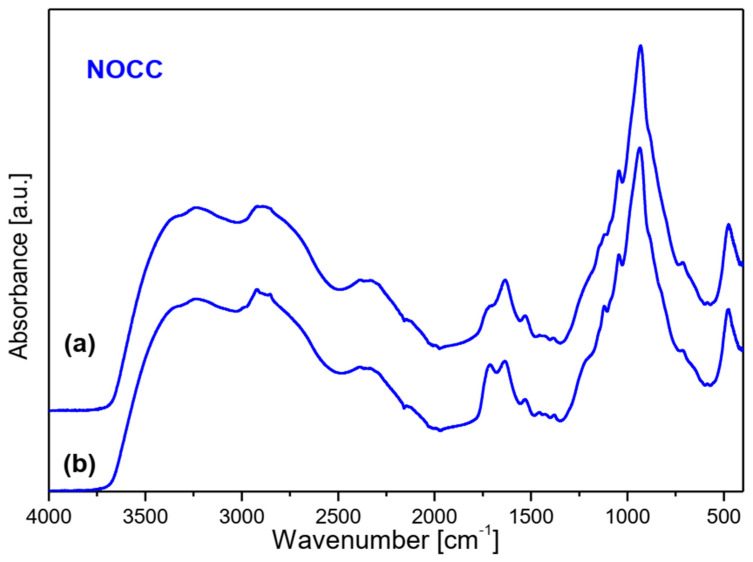
FTIR spectra of films produced by crosslinking of magnesium phytate with **OCC** and **NOCC** chitosan derivatives. **Top** layer (**a**) and **bottom** layer (**b**) of membrane.

To characterize the interaction between chitosan and phytate in crosslinked products the IR spectra of the synthesized films were measured. They are presented in Figure 6. They revealed bands characteristic for both these components [36,37,38]. The fundamental differences between the substrates and final products are observed in the 2000–3700 cm^−1^ range. A very strong and broad spectral pattern is observed in this range built of a clear triplet with maxima at about 3175–3234, 2870–2921 and 2330–2390 cm^−1^. Such a complex band is characteristic for strong hydrogen bonds O–H···O formed between the O–H group of phosphate from the phytate unit and C=O chromophore of chitosan, or, inversely, between the C=O group of phytate and H–O bond from chitosan. Such a triplet structure in this vibrational range is typical for Fermi resonance of the υ(O-H) and δ(OH) vibrations [39]. It should be noted that the HB formed in the films obtained from the **OCC** derivative are weaker that those from the **NOCC** derivative observed at 3234, 2917–2921 and 2334–2386 cm^−1^.

The bands originating from the vibrations of the phytate component are observed at 2873, 2324–2385, 2125–2139, 1627–1632, 1110–1120, 1020, 938–939 and 476–494 cm^−1^ in the IR spectra of films [37,38]. On the other hand, the bands typical for chitosan and its derivatives are observed in the ranges listed in Table S1A.

Very strong and broad bands observed for the films appear in the 600–1250 cm^−1^ range; such a band is also observed in the spectra of **OCC** and **NOCC** derivatives. These bands and those originating from the HB interactions as well as υ(C=O) vibrations at 1720 cm^−1^ should be considered as proof of the crosslinking process in the chitosan/phytate system.

The IR studies of the obtained membranes allowed to define the interaction between the crosslinked components. The strong hydrogen bonds O–H···O formed between the O–H group of phosphate from phytate unit and C=O chromophore of chitosan as well as between the C=O group of phytate and H–O bond from chitosan are responsible for the crosslinking process in the chitosan/phytate system. Such interactions lead to the aggregation of the adjacent chitosan chains formed the assembled structures as building blocks similar to those described by Lei et al. [40].

**Figure 7:**
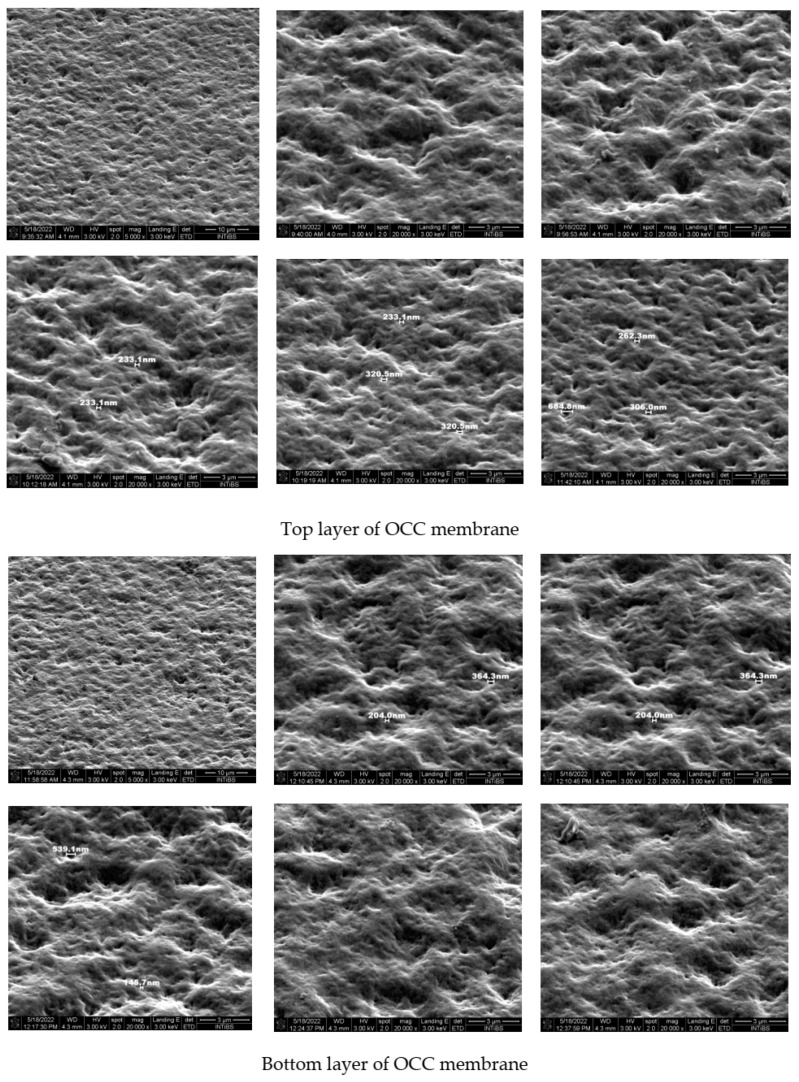
Photograph of the films recorded for **OCC** by electron microscope.

The surface morphology and tightness of the obtained membranes for **OCC** chitosan derivatives were studied using field emission scanning microscopy. Both sides of the films were studied to compare their properties. Figure 7 presents the results of these measurements.

Comparing the surface morphology of both sides of the membranes it is seen that they are similar. Regular unevenness is visible: depressions and elevations of similar depth (height). No pores or channels are extended into the bulk. This means that the components used for crosslinking interact effectively giving stable and uniform membranes. Both **OCC** and **NOCC** chitosan derivatives could be used for the production of good quality membranes. The final product exhibits good covering properties and could be used as a packaging material. The membrane is uniform because the water drop located on its surface does not penetrate the second side.

## 3. Materials and Methods

### 3.1. Materials

Chitosan of low viscosity (<200 mPa.s, 1% in acetic acid, CAS 9012-76-4) was obtained from Merck KGaA (Darmstadt, Germany). This product was previously characterized by the degree of deacetylation [36]. The degree of deacetylation (DD) for chitosan was 76%. 

Phytic acid (50 wt. % solution in water, d = 1.432 g/cm^3^), chloroacetic acid (99%, CAS 79-11-8), magnesium carbonate basic (tested according to Ph. Eur., heavy, CAS 39409-82-0), glyoxylic acid monohydrate (98%, CAS 563-96-2), sodium borohydride (≥98.0% powder, CAS 16940-66-2) and acetic acid (100%, glacial, anhydrous for analysis, CAS 16940-66-2) were purchased from Merck KGaA (Darmstadt, Germany). Sodium hydroxide (for analysis, CAS 1310-73-2), ethanol (96.0%, for analysis, CAS 64-17-5) and isopropanol (min. 99%, for analysis, CAS 67-63-0) were obtained from Chempur (Piekary Śląskie, Poland).

### 3.2. Synthesis of Magnesium Phytate

The phytate complexes were synthesized by changing phytic acid to a metal mole ratio. About 10 mL solution magnesium phytate was prepared dissolving 14.32 g (50%) IP6 and 0.91 g MgCO_3_ at room temperature for 2–3 h. After complexation, the content of the beaker was freeze-dried. The freeze-drying process was conducted by treating reaction products at −80 °C for 24 h, followed by drying them at pressure 0.02 mbar for 48 h using a M. Christ GmbH Alpha freeze-dryer.

### 3.3. Synthesis of Chitosan Derivatives

*N*-carboxymethyl chitosan (**NCC**) was synthesized using 1 g of chitosan (CC) dissolved in 100 mL of 0.15 mol aqueous acetic acid. It was treated with 4 g of solid glyoxylic acid and the obtained solution was mixed with magnetic stirrer. After storing for one hour, 3 g of solid sodium borohydride NaBH_4_ dissolved in 5 mL water was added to this solution. This mixture was treated by pure ethanol in a 1:1 volume ratio, which produced the precipitate which was filtered giving an **NCC** residue. It was washed with a small amount of water and three times with 70% ethanol. White powder was dried at 60 °C for 12 h.

*O*-carboxymethyl chitosan (**OCC**) was synthesized by soaking 2 g of chitosan in 30 mL NaOH solution for 3 h. Such a pre-treated chitosan was filtered and dissolved in 30 mL of 50% isopropanol by stirring it for 0.5 h. Independently, the second solution was prepared with 5 g of monochloroacetic acid dissolved in 7 mL of 50% isopropanol and added to the formed mixture. The obtained solution was stirred at 4 °C for 5 h, kept frozen for 24 h and filtered by washing with 70% ethanol. Separated powdered **OCC** was dried at 60 °C for 12 h.

*N*,*O*-carboxymethyl chitosan (**NOCC**) was synthesized from initially pre-soaked 2 g of chitosan with 20 mL of 20% solution prepared from sodium hydroxide. After 12 h this soaked chitosan was separated by filtration and placed in a two-necked flask in which 15 mL isopropanol was introduced. The resulting mixture was stirred using a magnetic stirrer for 0.5 h at room temperature. The separately-prepared 10 mL of isopropanolic solution with 1.43g of solid monochloroacetic acid was added to the flask with chitosan and stirred for 0.5 h. The content of the flask was refluxed for 3 h at 60 °C, filtered after 3 h and washed with 70% ethanol three times—the last time with isopropanol. The final **NOCC** residue was dried at 60 °C for 12 h.

The carboxymethylated chitosan derivatives were used for their crosslinking with magnesium phytate. This solution was used for crosslinking carboxymethylated chitosan derivatives which were prepared by dissolving of 0.04 g in 1 mL water. The **NCC** solution obtained in this way was fully transparent with pH = 8.73, but **OCC** and **NOCC** solutions were turbid showing pH: **OCC**—10.14 and **NOCC**—11.94, respectively. Their transparency grew gradually while adding some amount of 80% lactic acid. Adding 250 µL of the solution changed the pH of **OCC** solution to pH 5.02, but adding 300 µL of lactic acid to **NOCC** solution changed its pH to 5.14.

**Figure 8:**
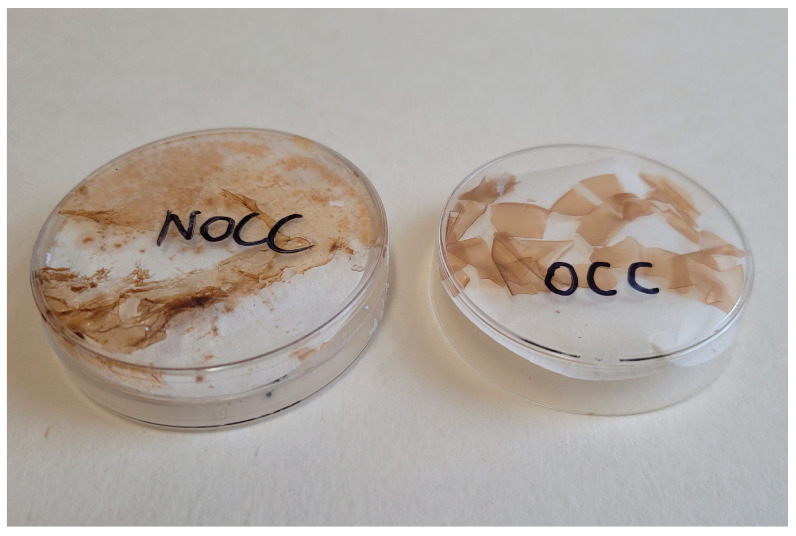
Photographs of the films obtained by crosslinking of the **OCC** and **NOCC** solutions by magnesium phytate.

The phytate/chitosan crosslinking chitosan-based membranes were produced in the form of thin films. The above-described mixtures of magnesium phytate and **OCC** and **NOCC** chitosan derivatives were cast in a Petri dish obtaining thin transparent films that were sprinkled using a diluted solution of magnesium phytate and kept in a desiccator containing silica gel. The photographs of the obtained films are shown in Figure 8.

The following conclusions can be drawn on the production process used for crosslinking of the chitosan derivatives: (1) the crosslinking for the **NCC** derivative does not occur; (2) among the studied chitosan derivatives, **OCC** is the most effectively crosslinked by magnesium phytate–the film formed by this substrate exhibits good mechanical parameters of strength, resistance and stability.

The obtained films were studied using scanning electron microscopy (SEM), IR and Raman measurements, as well as electron UV–Vis and emission spectra.

### 3.4. Spectroscopic Studies

IR spectra were measured using a Nicolet iS50 FT-IR (Thermo Fisher Scientific Inc., Waltham, MA, USA) spectrometer equipped with an Automated Beamsplitter exchange system (iS50 ABX containing DLaTGS KBr detector and DLaTGS Solid Substrate detector for mid-IR and far-IR regions, respectively) and a built-in all-reflective diamond ATR module (iS50 ATR). Polycrystalline mid-IR spectra were collected in the 4000–400 cm^−1^ range in KBr pellets and far-IR spectra in 600–50 cm^−1^ in Nujol mull. Spectral resolution was set to 2 cm^−1^.

Raman spectra in the 4000–80 cm^−1^ range were measured in back scattering geometry with a FT Bruker 110/S spectrometer (Bruker Corporation, Billerica, MA, USA). The resolution was 2.0 cm^−1^. The YAG:Nd (excitation wavelength 1064 nm) laser was used as an excitation source.

Room temperature electron absorption spectra were measured in the 200–1500 nm spectral range using a Cary-Varian 5E UV–Vis-near-IR spectrophotometer (Agilent Technologies, Inc., Santa Clara, CA, USA). Diffuse reflectance spectra were recorded with a Praying Mantis diffuse reflectance accessory (Harrick Scientific Products, Inc., Pleasantville, NY, USA). In these measurements the baseline was first recorded for Al_2_O_3_ powder, and next this line was subtracted from that obtained for particular powder sample spectra.

Decay profiles and emission spectra were recorded with a grating spectrograph (Princeton Instr. Model Acton 2500i, Princeton Instruments, Trenton, NJ, USA) coupled to a streak camera (Hamamatsu Model C5680, Hamamatsu Photonics Europe GmbH, Herrsching, Germany). For excitation, a femtosecond laser (Coherent Model “Libra”) was used. The laser delivers a train of 89 fs pulses at a wavelength of 800 nm and a pulse energy of 1 mJ, with repetition rates regulated up to 1 kHz. To attain light pulses at different wavelengths the laser was coupled to an optical parametric amplifier (Light Conversion Model OPerA) that can operate in the range of 230–2800 nm.

The surface morphology of the foil samples (both sides) was studied with a field emission scanning (FE-SEM) microscope (FEI NovaNanoSEM 230, FEI Company, Hillsboro, OR, USA). A secondary electron (SE) detector was applied for imaging, and low beam energy (3 keV) was used to avoid charging.

## 4. Conclusions

Novel chitosan/magnesium phytate membranes were obtained using highly deacetylated chitosan and its *N*-carboxymethyl (**NCC**), *O*-carboxymethyl (**OCC**) and *N*,*O*-carboxymethyl (**NOCC**) derivatives. **OCC** crosslinked by magnesium phytate proved to be the most effective. The films that form on this substrate exhibit good mechanical parameters of strength, resistance and stability. The spectroscopic studies of the obtained membranes showed that the strong O–H···O hydrogen bonds formed between the OH and C=O groups of phytate and chitosan are responsible for the crosslinking process and strong interactions between these components. It was found that new type membranes produced from chitosan and magnesium phytate are mechanically stable and morphologically homogenous and uniform. They do not have pores or channels and, due to their good covering properties, are proposed as packaging materials. The membrane is impermeable to water, which does not penetrate the second side of the foil.

## Figures and Tables

**Table 1:** The triplet states in the studied compounds.

	T_1_	T_2_	T_3_
Chitosan	19,607	21,834	23,310 cm^−1^
**NCC**	20,000	22,573	23,310 cm^−1^
**OCC**	20,408	21,276	22,471 cm^−1^
**NOCC**	20,408	21,691	23,255 cm^−1^

## Data Availability

Not applicable.

## Supplementary Materials

The following supporting information can be downloaded at: https://www.mdpi.com/article/10.3390/molecules28165987/s1, Table S1. A. Assignment of the bands observed in the MIR spectra. B. Assignment of the bands observed in the Raman spectra.

## Sample Availability

Samples of the compounds (*N*-carboxymethyl, *O*-carboxymethyl, and *N*,*O*-carboxymethyl) are available from the authors.
