# Supplementary material for: Chitosan and Its Carboxymethyl-Based Membranes Produced by Crosslinking with Magnesium Phytate
Source: Molecules. 2023 Aug 10;28(16):5987. doi: 10.3390/molecules28165987 (PMC10459599; doi:10.3390/molecules28165987)
Supplement: Supplementary file 1 [file molecules-28-05987-s001.zip › molecules-2449782-supplementary.pdf]

Table S1A. Assignment of the bands observed in the MIR spectra.

Chitosan	NCC	NOCC	OCC	Assignment of the bands
3440sh,3417sh	3523sh,3482sh	3344m	3439sh	υ(OH)$_{HB}$
3386vs,3352vs	3346m,3249sh 3213sh	3288m	3356m, 3285m	υ(NH_2)
3188sh, 3071sh	3107sh	3175sh	3183sh	υ(NH_2)
	3055sh	3085sh	3092sh	υ(NH)
2913m	2924sh	2913sh	2909sh	υ$_{as}$(CH_3)
2872m	2875sh	2864m	2867m	υ$_s$(CH_3)
1646s	1657w		1649w	υ(CO)
1587s				υ(NH_2)
	1590w	1593m	1591w	υ(COO)
1565sh, 1543 sh				υ(NH_2)
1455sh	1458m	1445sh	1455sh	δ(CH)+ω(CH_2)+υ(OH)
1418m	1424m	1424vs	1425vs	δ(CH)+δ(OH)
		1390sh	1414s	υ(COO)
1375m	1381m		1377sh	δ(CH_2)+δ(CH)+δ(OH)+υ(ϕ)
1319m	1340s	1319w	1319w	υ(CN)+δ(CH)
	1272m	1262sh	1283 sh	υ(CCOO)
1266m	1255m 1223sh	1266sh	1300sh 1264sh	δ(OH···O)+υ(C-C)+υ(C-O)+δ(CH)+ϱ(CH_3)
1150s	1159w	1145sh	1150w	υ(C-O-C)+υ(ϕ)+υ(C-OH)+υ(C-CH_3)+ϱ(CH_2)+ϱ(CH_3)
	1130m			υ(CH_2-C)
1061vs	1077w	1062m	1062m	υ(C-O-C)+υ(ϕ)+υ(C-OH)+υ(C-CH_3)+ϱ(CH_2)+ϱ(CH_3)
1025vs	1026m	1020m	1024s	υ(ϕ)+δ(CH)
993vs	995vs	997m	991m	υ(ϕ)+δ(CH)
944sh	943s		940sh	υ(C-N)
893m	883vw	879m	880m	υ(ϕ)+ϱ(CH_2)
	822s			υ(ϕ)+ϱ(CH_2)
	773w			υ(ϕ)+ϱ(CH_2)
703sh	707w	701w	701vw	ω(NH_2)+δ(ϕ)
698vw	694sh	695w	694vw	δ(COO)
666sh 660m	676vw	654w	661w	γ(OH···O), δ(ϕ)
557m	575 vw	574w 565w	564w	γ(NH)+γ(C-C=O)+ω(CH_3)
518m	524vw	518vw 512vw	523vw	δ(ϕ)+ω(CH_3)
	461vw			γ(OH)+γ(ϕ)

ϕ - pyranoid ring; υ - stretching; δ - in-plane bending; γ, ω - out-of-plane bending, HB – hydrogen bond.

Table S1B. Assignment of the bands observed in the Raman spectra.

**Chitosan**	**NCC**	**NOCC**	**OCC**	**Assignment of the bands**
3362w				$\upsilon(OH)_{HB}$
3308w				$\upsilon(OH)_{HB}$
2932vs	2929w	2940m<br>2916m	2930s	$\upsilon_{as}(CH_3)$
2885vs	2895m	2896s	2888vs	$\upsilon(CH_2)$
2818sh		2812vw		$\upsilon_s(CH_3)$
2743w		2715vw	2731vw	$\upsilon(CH)$
1654w		1656vw	1657vw	$\upsilon(CO)$
1591m	156vw<br>1528vw	1598w	1596vw	$\delta(NH_2)$ and $\upsilon(COO)$
1458m	1460w	1462vw<br>1445vw	1461w	$\delta(CH)+\omega(CH_2)+\delta(OH)$
1411m	1400w	1414w	1411w	$\delta_s(CH_3)+\delta(CH)$
1377vs	1375m	1376m	1376m	$\delta(CH_2)+\delta(CH)+\delta(OH)+\upsilon(\phi)$
1325m	1345sh	1327vw		$\upsilon(CN)+\delta(CH)$
1263m	1264w	1261w	1261w	$\delta(OH\cdots O)+\upsilon(C\text{-}C)+\upsilon(C\text{-}O)+\delta(CH)+\varrho(CH_3)$
1146m	1149sh	1147w	1150w	$\upsilon(C\text{-}O\text{-}C)+\upsilon(\phi)+\upsilon(C\text{-}OH)+\upsilon(C\text{-}CH_3)+\varrho(CH_2)+\varrho(CH_3)$
1114s	1113w	1115m	1115m	$\upsilon(C\text{-}O\text{-}C)+\upsilon(\phi)+\upsilon(C\text{-}OH)+\upsilon(C\text{-}CH_3)+\varrho(CH_2)+\varrho(CH_3)$
1093s	1087sh	1080vs	1080vs	$\upsilon(C\text{-}O\text{-}C)+\upsilon(\phi)+\upsilon(C\text{-}OH)+\upsilon(C\text{-}CH_3)+\varrho(CH_2)+\varrho(CH_3)$
1044sh	1073sh	1070m<br>1042sh	1072m<br>1045w	$\varrho(CH_3)+\delta(CH)+\delta(OH)$
991vw	993vw		994vw	$\upsilon(\phi)+\delta(CH)$
936w				$\upsilon(C\text{-}N)$
896m	901vw<br>887w	905m<br>893sh	898m	$\upsilon(\phi)+\varrho(CH_2)$, $\delta(COO)$
784w<br>768w	768vs	785w	788vw<br>778vw	$\omega(NH_2)+\delta(\phi)$
703w	696vw	701w	701w	$\gamma(OH\cdots O)$
606w	592w<br>579vs	596vw		$\delta(\phi)$, $\omega(COO)$
566sh				$\gamma(NH)+\gamma(C\text{-}C{=}O)+\omega(CH_3)$
 493m		517vw<br>493vw	 491w	$\delta(CO\text{-}NH)+\delta(C\text{-}CH_3)$
479m			477vw	$\delta(COC)$
444m, 424m	465m<br>406w	421vw	448vw<br>425w	$\gamma(OH)+\gamma(\phi)$. $\varrho(COO)$
370sh	391w	373w	373w<br>362w	$\gamma(OH)+\gamma(\phi)$
357m<br>330m	345w<br>323sh	358w<br>330w	354w<br>290vw	$\gamma(OH)+\gamma(\phi)$, $\gamma(COO)$
285m	276vw	273w	278w	$\delta(C\text{-}MH\text{-}C)+\gamma(OH)$
216m	206vw	195vw	195vw	$\delta(C\text{-}MH\text{-}C)+\gamma(OH)$

ϕ - pyranoid ring; υ - stretching; δ - in-plane bending; γ, ω - out-of-plane bending, HB – hydrogen bond.
